# Questionnaire survey on pharmacists’ roles among non- and health care professionals in medium-sized cities in Japan

**DOI:** 10.1038/s41598-023-32777-0

**Published:** 2023-04-04

**Authors:** Fukuko Horio, Tokunori Ikeda, Yanosuke Kouzaki, Tomoo Hirahara, Kengo Masa, Sawana Narita, Yusuke Tomita, Shu Tsuruzoe, Akihiko Fujisawa, Yuki Akinaga, Yoko Ashizuka, Yuki Inoue, Ayaka Unten, Katsutoshi Okamura, Yuiko Takechi, Yasuhiro Takenouchi, Fuka Tanaka, Chiharu Masuda, Yusuke Sugimura, Yuji Uchida

**Affiliations:** 1 Laboratory of Clinical Pharmacology and Therapeutics, Faculty of Pharmaceutical Sciences, Sojo University, 4-22-1, Ikeda, Kumamoto 860-0082 Japan; 2 Department of Neurology, National Hospital Organization Kumamoto Medical Center, Kumamoto, Japan; 3 Department of Neurology, Uki General Hospital, Kumamoto, Japan; 4 Department of Pharmacy, Kumamoto University Hospital, Kumamoto, Japan; 5 Department of Respiratory Medicine, Graduate School of Medical Sciences, Kumamoto University, Kumamoto, Japan; 6 Musashigaoka Hospital, Kumamoto, Japan; 7 Kumamoto Dermatology, Plastic Surgery Clinic, Kumamoto, Japan; 8 Department of Neurology, Sugimura Hospital, Kumamoto, Japan

**Keywords:** Health care, Public health

## Abstract

Although the scope of pharmacists’ work has expanded in Japan, people’s perception of this is unclear. To contribute to medical care together with non- and health care professionals, clarifying the perceptions of these groups is important to best utilize pharmacist professionals. We conducted a cross-sectional questionnaire survey among non-health care professionals (n = 487) and nurses (n = 151), medical doctors (n = 133), and pharmacists (n = 204) regarding the work of pharmacists. The questionnaire comprised 56 items in four categories associated with the roles of pharmacists. For each questionnaire item, we performed logistic regression analysis to compare pharmacists’ opinions with those of other professionals and non-health care professionals. Opinions were similar between pharmacists and nurses or medical doctors regarding “collecting patient information” and “providing drug information to patients.” However, there were differences in perceptions regarding “medical collaboration” (nurses; 8/23 items, physicians; 11/23 items) and “community medicine” (nurses; 9/15 items, physicians; 11/15 items), and pharmacists themselves perceived greater roles related to health care collaboration and community health care. Perceptions of non-health care professionals were poorer than those of pharmacists in all categories (47/56 items). These results suggest that pharmacists must actively communicate to help others understand their specialty and build trusting relationships to improve patient care.

## Introduction

In Europe during the 2010s, it has been reported that electronic prescribing and robotic dispensing have reduced the workload of pharmacists and decreased the number of dispensing errors^1–3^. The greater use of mechanization is expected to have an increasing impact on pharmacists’ work worldwide^4^.

The situation in Japan has been different in some respects. For instance, in many countries, pharmacy technicians are common. These professionals assist pharmacists^5^ and are in charge of various operations centered upon physical work. However, pharmacists in Japan have roles involving interpersonal work, such as auditing prescriptions and explaining dispensed medicines, as well as physical work, such as inventory control and measuring prescription medicines. As a result, pharmacists in Japan are less able to concentrate on interpersonal work than pharmacists in other countries. Therefore, mechanization is expected to have an impact on interpersonal work among Japanese pharmacists.

The rapid progression of population aging^6^ and prolongation of the mean lifespan^7^ in Japan may lead to a new role for pharmacists. Because medical expenses have and will continue to escalate^8^, the Japanese government is promoting self-medication according to the World Health Organization definition to help control costs^9,10^. Self-medication means that patients obtain their medicines at a pharmacy or drugstore without consulting with a medical doctor. In fact, Japan has a tax deduction system, called the “self-medication tax system,” for medical expenses related to the use of over-the-counter drugs^11^. In some counties, self-medication is well developed^12–14^, and pharmacists in these countries serve as the first point of contact for non-health care professionals for patient treatment and consultation^9^. In other words, it is expected that Japanese pharmacists also actively communicate with patients, similar to other countries where self-medication is common.

The increased proportions of older people and people living alone^15^ highlight the importance of home-based medical care provided by Japanese health care professionals. Older people have difficulty visiting a hospital owing to decreased motor function or no means of transportation; thus, the demand for home medical care has been increasing. Japanese health care professionals provide proper home medical care to homebound patients and those who require care at home. These health care professionals mainly comprise medical doctors, nurses, and pharmacists. Medical doctors and nurses expect pharmacists to supply and manage medicinal agents and provide instructions for medication adherence to homebound patients^16–19^. In fact, it has been reported that collaboration between the pharmacist and other medical professionals has numerous benefits for patients (e.g., in the management of chronic disease, such as timely detection of a duplicate dose, early detection of adverse effects, improved adherence, and early achievement of treatment goals)^20,21^. Moreover, prescription refill systems have reduced the burden on medical doctors and have improved patient care in some countries^22,23^; these systems have been also implemented in Japan from 2022^24^. Thus, medical collaboration with pharmacists is an important factor in the provision of high-quality health care, and the pharmacist profession in Japan is changing. However, there are few reports concerning how this profession is perceived by others^25^. Furthermore, studies of how pharmacists perceive their own scope of work are similarly scarce^26,27^. To contribute to medical care provided by non-health care and health care professionals and to collaborate with health care professionals, clarifying the perceptions of these groups is important in considering how to best utilize pharmacist professionals.

Against this background, we conducted a questionnaire survey on current opinions regarding the scope of work required of pharmacists in Japan from the perspective of non-health care professionals and health care professionals (nurses, medical doctors, and pharmacists themselves).

## Methods

### Study design

This was a cross-sectional, exploratory, observational study and questionnaire survey.

### Study tool

We developed a questionnaire to investigate opinions regarding pharmacists’ roles, using the following method. Only one study that included a questionnaire survey on opinions about the work of pharmacists among non-health care professionals and pharmacists has been previously reported in Japan^27^. Referencing that study, the questionnaire items were designed by nine fourth-year pharmacy students, a pharmacist, two medical doctors, and a biostatistician. Specifically, we used an approach with reference to the Delphi method^28^, as follows. In the first step, the pharmacy students independently created the basic questionnaire items. In the next step, the students were randomly divided into three groups to discuss, verify, reconstruct, and aggregate basic questionnaire items. After group discussion, a team meeting was held in which questionnaire items presented by each group were discussed, and modified questionnaire items were generated. The second and third steps were repeated 11 times until consensus among all participants was reached regarding the finalized questionnaire. Additionally, the content of the developed questionnaire was checked by another two pharmacists and revised again. The final questionnaire was divided into four main categories:

- Category 1: patient information collected by pharmacists,
- Category 2: information that pharmacists provide to patients,
- Category 3: information that pharmacists communicate to medical doctors and nurses,
- Category 4: pharmacists’ engagement in community health care.

Responses to each question had five options, and we constructed dummy variables corresponding to these options, as follows: score of 1 = completely disagree, 2 = somewhat disagree, 3 = unsure, 4 = somewhat agree, and 5 = completely agree.

We also collected information regarding participants’ age (20–30 s/40 s–50 s/60 s +) and sex. For health care professionals, we queried the years of experience in each health occupation (< 5 years/ < 10 years/ ≥ 10 years/ ≥ 20 years/ ≥ 30 years/ ≥ 40 years), the number of beds in the hospital or clinic where they worked (≤ 19/20–99/100–199/200–399/ ≥ 400), and whether there were pharmacists working in their hospital or clinic.

From March 2021 to October 2021, questionnaire surveys in paper form were distributed to health care professionals, which included nurses, medical doctors, and pharmacists. In recruiting health care professionals, we selected pharmacies, hospitals, and clinics in Kumamoto, Kagoshima, and Nagasaki in Kyushu, where pharmacy students at Sojo University completed clinical training; medical care in these clinics reflects the state of medicine in medium-sized cities of Japan. We asked head pharmacists and clinic directors in these locations whether they would agree to cooperate with our survey, and those who agreed were included in the study. We obtained questionnaire responses from 151 nurses, 133 medical doctors, and 204 pharmacists.

For non-health care professionals, we used a professional Internet research company (ASMARQ Co., Ltd., Tokyo, Japan). The company conducts market research, including academic research, and has access to approximately 16 million Japanese panels and recruits from these according to the needs of each study. Non-health care professionals were recruited on the condition that living standards and the medical environment of their residential area must be similar to those of health care professionals (Kumamoto, Kagoshima, and Nagasaki prefectures). We used a research company because it would have been challenging for the researchers to identify a wide range of individuals who met these detailed criteria. We also engaged the research company to conduct the self-designed questionnaire on our behalf. Each respondent was asked to consent to the survey as part of the questionnaire, and only those who consented were administered the survey.

In January 2021, ASMARQ distributed the questionnaire online and collected completed surveys that had no missing data. ASMARQ screened 784 registered panels according to the above criteria, and 500 individuals who were eligible completed the questionnaire. We checked and discussed the questionnaire results of all participants; responses for 13 participants were excluded owing to inappropriate answers, such as repeated responses. Finally, we included completed questionnaire surveys from 487 non-health care professionals in this study.

### Eligibility criteria

The eligibility and exclusion criteria were as follows. For health care professionals, the inclusion criteria were: (1) Japanese nationality; (2) working as a nurse, medical doctor, pharmacist; and (3) living in Kumamoto, Kagoshima, or Nagasaki in the Kyushu region of Japan (Supplementary Fig. S1). The exclusion criteria were: (1) living in the Okinawa region or isolated islands in Kyushu, Japan (i.e., island areas); and (2) living in Fukuoka and Kitakyushu (i.e., cities larger than Kumamoto city).

For non-health care professionals, the inclusion criteria were: (1) respondents registered in an AAMARQ panel; (2) Japanese nationality; aged ≥ 20 and ≤ 80 years; and (3) living in Kumamoto, Kagoshima, or Nagasaki in Kyushu, Japan. The exclusion criteria were: (1) the respondent or their family worked in medical facilities such as a hospital, clinic, dental office, pharmacy, maternity home, or long-term care health facility; (2) living in the Okinawa region or isolated islands in Kyushu, Japan; (3) living in Fukuoka and Kitakyushu (i.e., cities larger than Kumamoto city); and (4) living in a town or village (population of fewer than 50,000 inhabitants).

### Ethical considerations

Written informed consent was obtained from all participants after they had received a full explanation of the questionnaire (paper or web-based). Additionally, participants’ information was acquired in such a way that specific individuals could not be identified. Our study was conducted according to the Declaration of Helsinki and the ethical guidelines for Medical and Health Research Involving Human Subjects of the Ministry of Health, Labour and Welfare of Japan. The study protocol was approved by the institutional review board of Sojo University, Faculty of Pharmaceutical Sciences (Permit Number: 2020-3).

### Statistical analysis

For categorical variables, we performed a pairwise Fisher’s exact test with Bonferroni correction. In the analysis between pharmacists and non-health care professionals, certain variables, as covariates, were not applicable to non-health care professionals, such as years of experience, number of beds in the facility, and presence or absence of pharmacists in the hospital or clinic, unlike nurses and medical doctors. These variables are specific to health care professionals and are important covariates in comparisons between pharmacists and nurses or medical doctors. Thus, binomial logistic regression analysis was performed to discriminate between pharmacists and non-health care professionals in estimating the odds ratio (OR) of each questionnaire item separately from the analysis between pharmacists and nurses or medical doctors. The age and sex of non-health care professionals can influence an increase in hospital visits and the types of related disorders^29–31^, respectively, and may provide opportunities to come into contact with pharmacists. Therefore, the perception of non-health care professionals regarding pharmacists’ works may be affected. Thus, we selected categorical variables age and sex as covariates in the model.

In comparison between pharmacists and nurses or medical doctors for each questionnaire item, we used multinomial logistic regression analysis. Because it is possible that years of experience, hospital size, and presence or absence of a pharmacist influence the perception of pharmacist’s work among nurses or medical doctors, we selected age, sex, years of experience, number of beds in the facility, and presence or absence of pharmacists in the hospital or clinic as covariates. In this model, we included sex and the presence or absence of pharmacists in the hospital or clinic as categorical variables; other variables were included as continuous variables. This was an exploratory observational study, and we did not conduct adjustment for multiplicity. There were fewer than 5% missing values, so we performed a complete case analysis^32^. The analyses were performed using R version 4.0.3 (The R Foundation for Statistical Computing, Vienna, Austria), and we used the forester package (https://github.com/rdboyes/forester) in R version 4.0.3 (https://cran.r-project.org/bin/windows/base/old/4.0.3/) to create the figures. The level of statistical significance was set at p < 0.05.

### Sample size

This was an exploratory observational study, so we did not design the sample size for hypothesis testing in advance. In this regard, 975 questionnaire survey data items (487 non-health care professionals, 151 nurses, 133 medical doctors, and 204 pharmacists) were used in the analysis. Because approximately 10 or greater events per covariate are required for logistic regression^33^, explanatory variables were appropriately selected according to the sample size, with this in mind.

## Results

### Participant characteristics

We analyzed data of 204 pharmacists, 487 non-health care professionals, 151 nurses, and 133 medical doctors. The characteristics of each group are shown in Table 1. The number of years of experience in each job category ranged from fewer than 5 years to more than 40 years (Supplementary Fig. S1).

**Table 1:** Characteristics of participants.

		Pharmacists	Non-health care professionals	Nurses	Medical doctors	*p*-value		
		n = 204	n = 487	n = 151	n = 133	Pharmacists vs. Non-health care professionals	Pharmacists vs. Nurses	Pharmacists vs. Medical doctors
Age, years	20 s–30 s	123	75	96	43	NA	0.35	NA
	40 s–50 s	73	241	47	69			
	60 s +	9	171	7	20			
Sex	men	84	306	13	106	< 0.001	< 0.001	< 0.001
	women	120	181	138	27			
Years of work experience	< 5	51	–	25	8	–	NA	NA
	< 10	57		34	19			
	≥ 10	50		47	45			
	≥ 20	29		19	32			
	≥ 30	13		20	20			
	≥ 40	4		4	8			
Number of facility beds	≤ 19	101	–	9	5	–	< 0.001	0.14
	20–99	0		0	0			
	100–199	23		69	38			
	200–399	9		21	18			
	≥ 400	71		52	72			
Pharmacists (including part-time) employed at the hospital	Yes	103	–	142	128	–	0.036	0.21
	No	0		9	5			

### Category 1: Opinions regarding the role of pharmacists in collecting patient information (Figs. 1 and 2, Supplementary Table S1)

**Figure 1:**
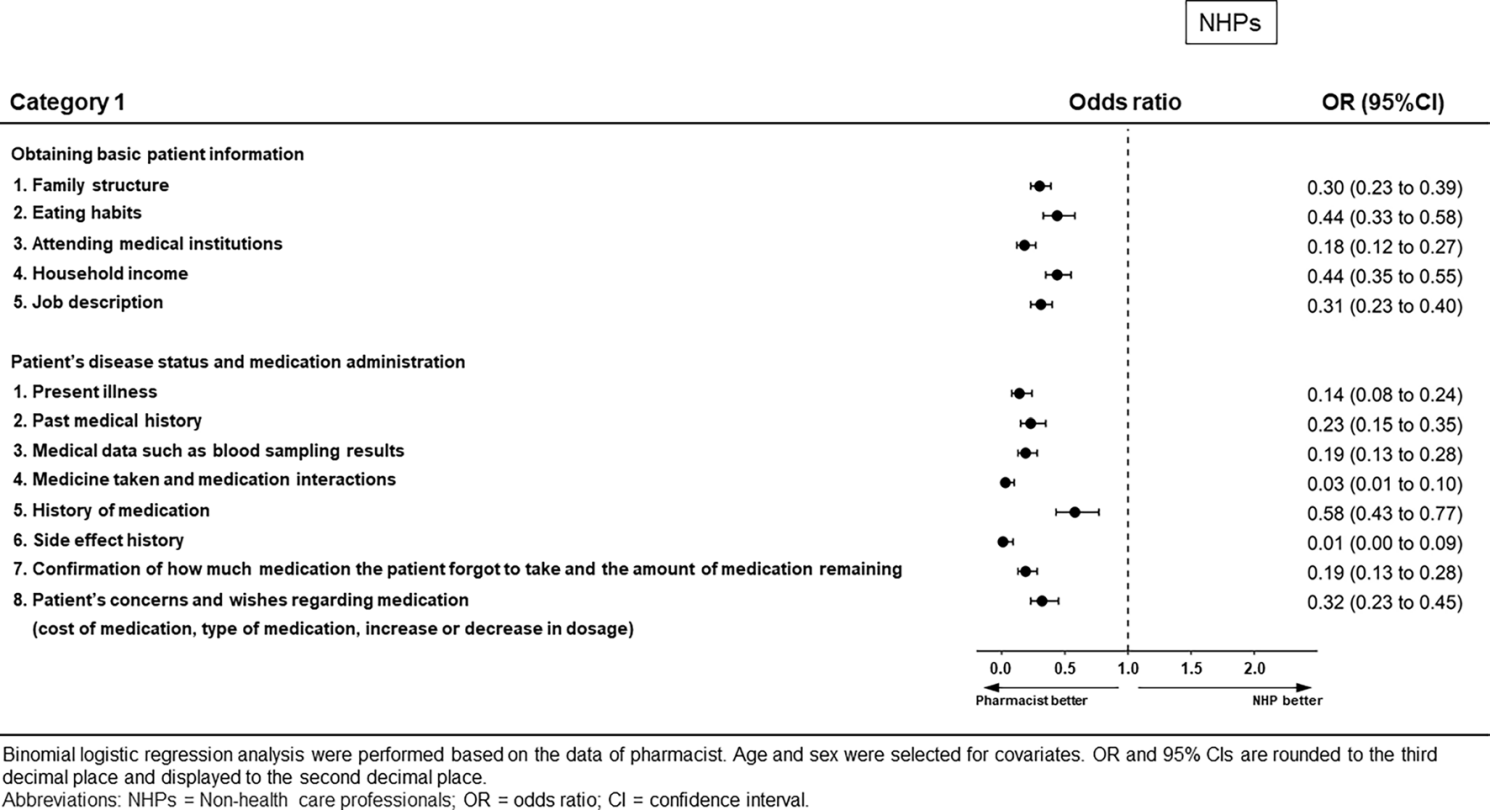
Results of binomial logistic regression analysis for Category 1 for non-health care professionals.

**Figure 2:**
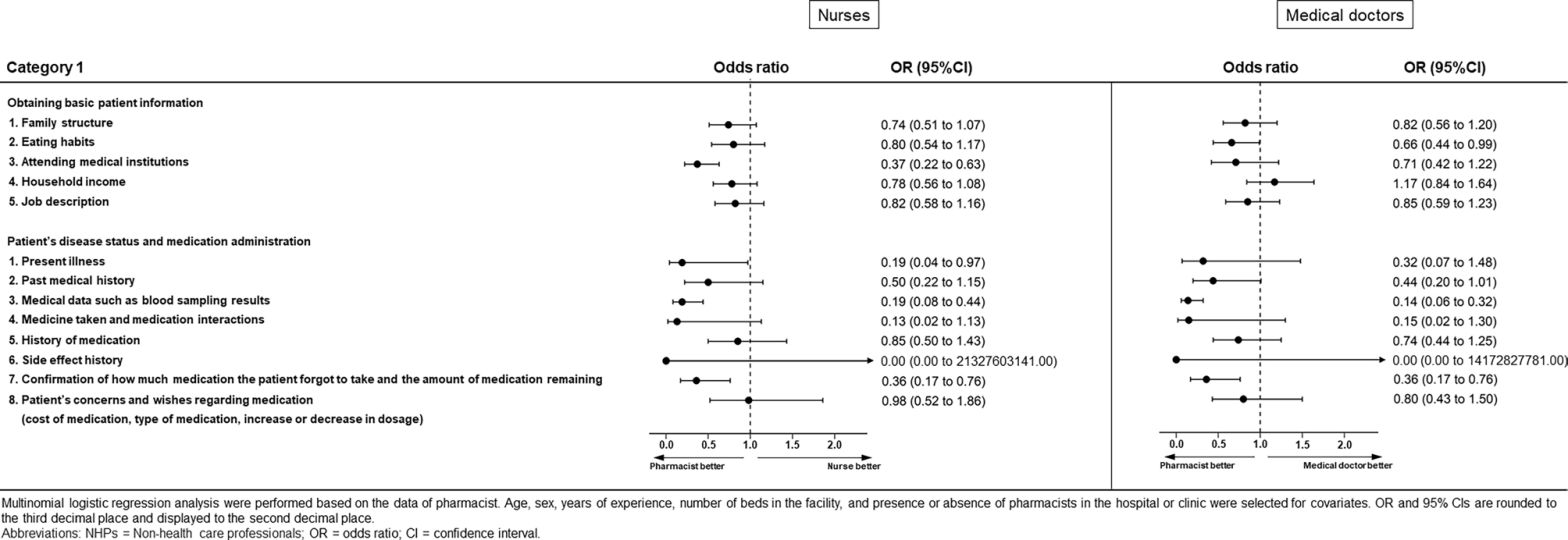
Results of multinomial logistic regression analysis for Category 1 for nurses or doctors.

Opinions with respect to pharmacists’ role in collecting patient information showed substantial differences between pharmacists and non-health care professionals in all questionnaire items. Pharmacists were more likely than non-medical professionals to perceive that patient information should be collected by pharmacists.

Although the opinions about this role were similar among pharmacists, nurses, and medical doctors, there were differences in some items. The ORs and 95% confidence interval (CIs) for which we found significant differences were as follows. For nurses, these were “attending medical institutions” (OR 0.37, 95% CI 0.22–0.63, *p* < 0.001), “present illness” (OR 0.19, 95% CI 0.04–0.97, *p* = 0.046), “medical data such as blood sampling results” (OR 0.19, 95% CI 0.08–0.44, *p* < 0.001), and “confirmation of how much medication the patient forgot to take and the amount of medication remaining” (OR 0.36, 95% CI 0.17–0.76, *p* = 0.01). For medical doctors, these were “eating habits” (OR 0.66, 95% CI 0.44–0.99, *p* = 0.045), “medical data such as blood sampling results” (OR 0.14, 95% CI 0.06–0.32, *p* < 0.001), and “confirmation of how much medication the patient forgot to take and the amount of medication remaining” (OR 0.36, 95% CI 0.17–0.76, *p* = 0.007).

### Category 2: Opinions regarding the role of pharmacists in providing drug information to patients (Figs. 3 and 4, Supplementary Table S2)

**Figure 3:**
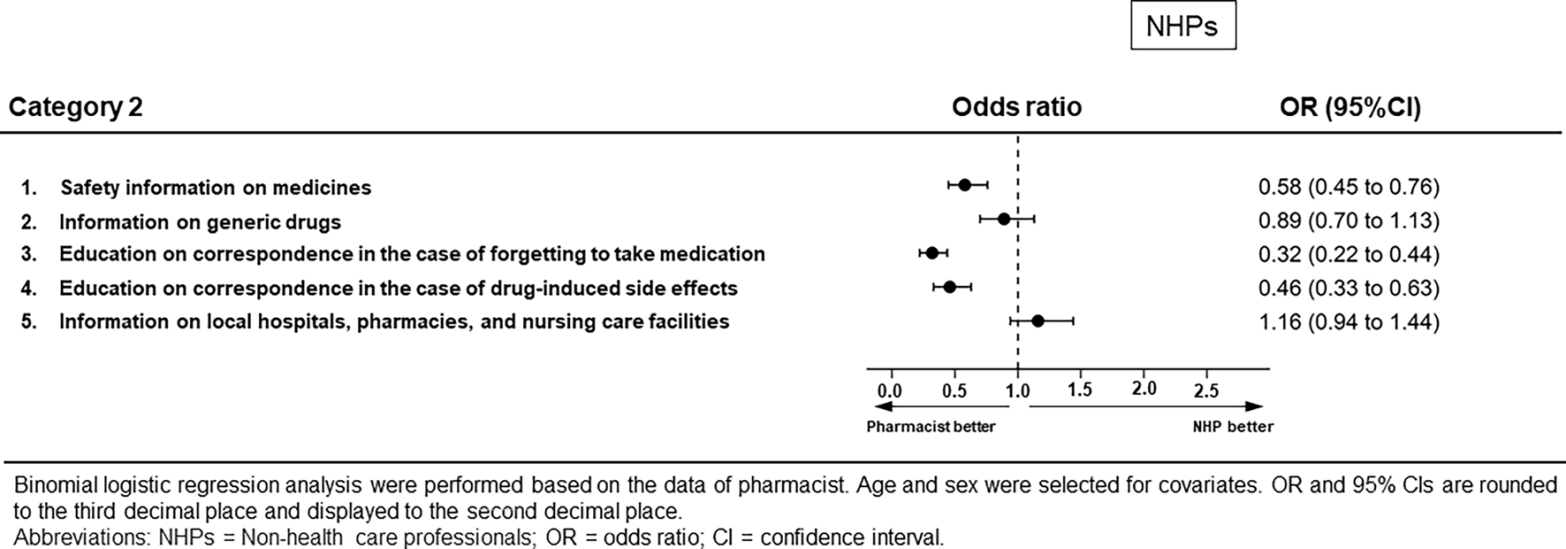
Results of binomial logistic regression analysis for Category 2 for non-health care professionals.

**Figure 4:**
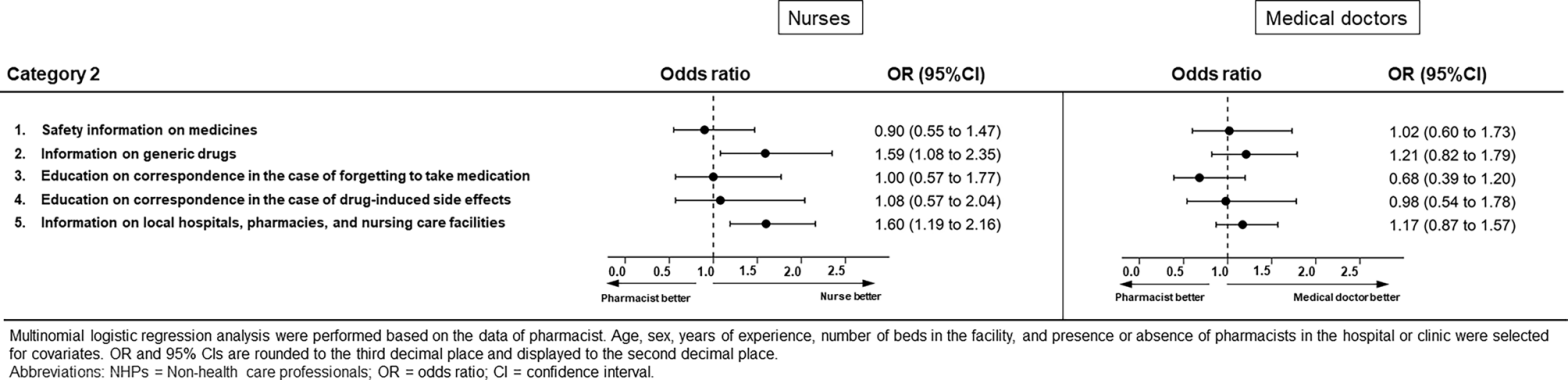
Results of multinomial logistic regression analysis for Category 2 for nurses or doctors.

Pharmacists were more likely than non-medical professionals to perceive that drug information should be provided by pharmacists, except regarding two questionnaire items (“generic drugs” and “local hospitals, pharmacies, and nursing care facilities”).

In contrast, nurses were more likely than pharmacists to perceive that this information should be provided by pharmacists. The ORs and 95% CIs for which we found significant differences were “generic drugs” (OR 1.59, 95% CI 1.08–2.35, *p* = 0.018) and “local hospitals, pharmacies, and nursing care facilities” (OR 1.60, 95% CI 1.19–2.16, *p* = 0.002). There was no difference in opinions regarding this role between pharmacists and medical doctors.

### Category 3: Opinions regarding the role of pharmacists in communication with doctors and nurses (Figs. 5 and 6, Supplementary Table S3)

**Figure 5:**
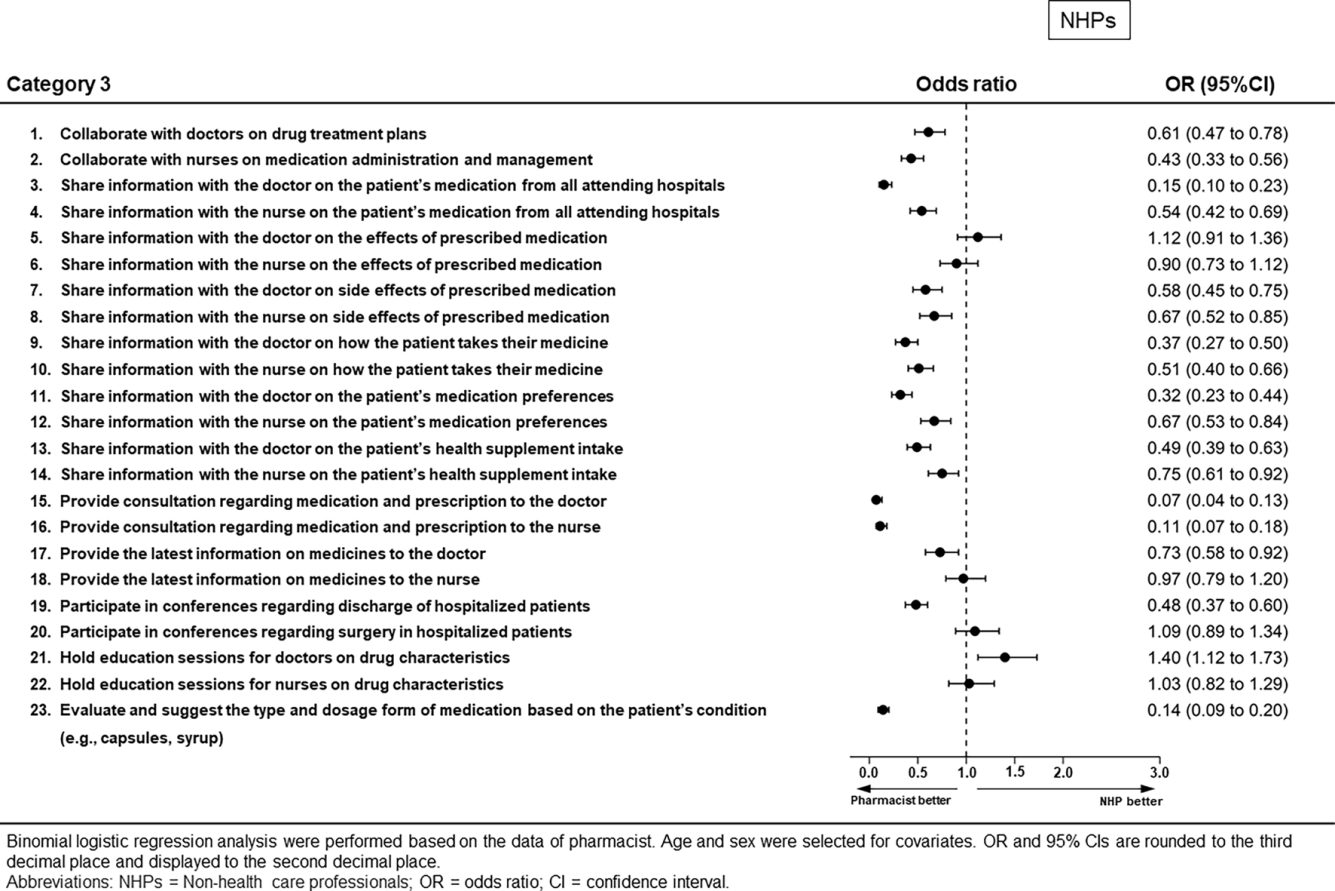
Results of binomial logistic regression analysis for Category 3 for non-health care professionals.

**Figure 6:**
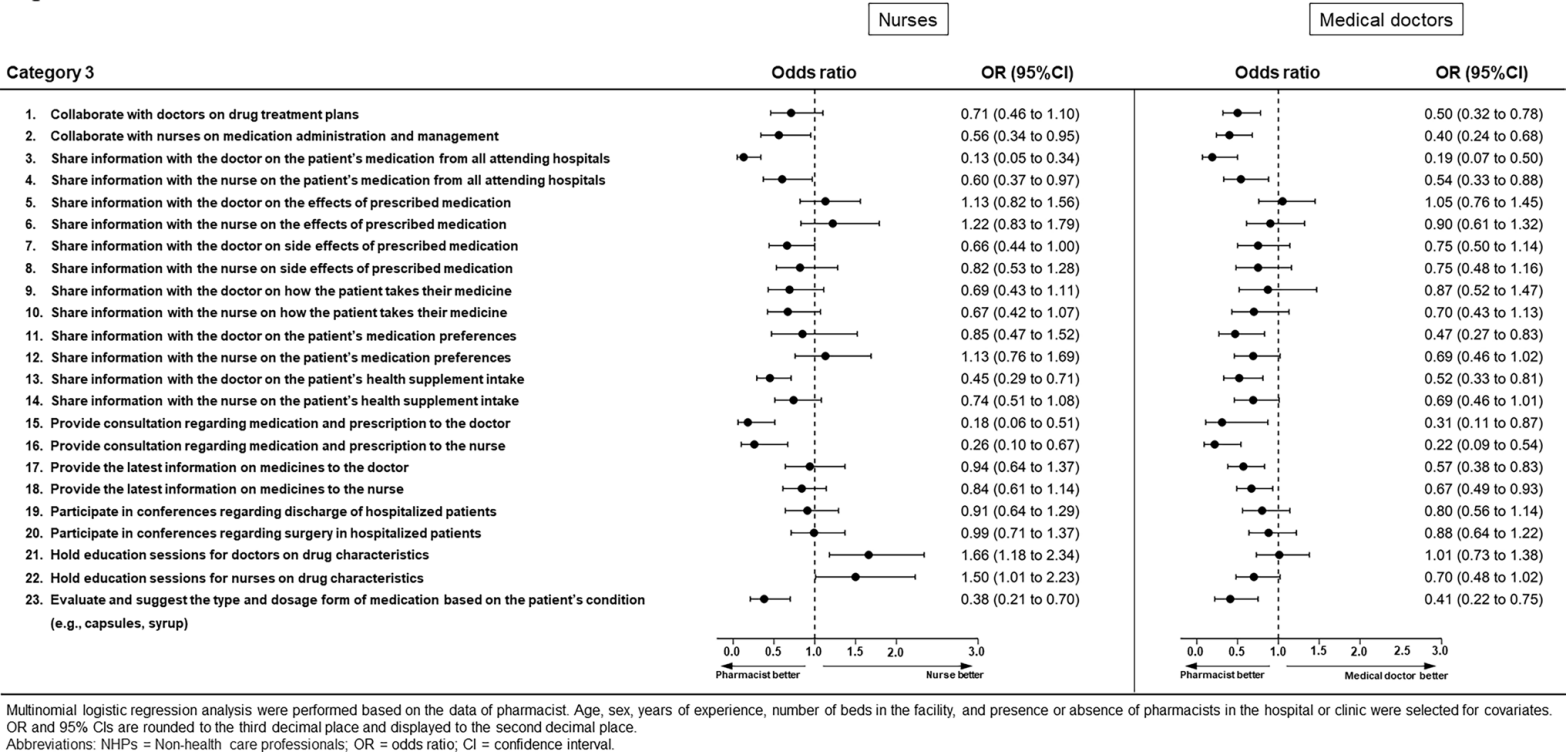
Results of multinomial logistic regression analysis for Category 3 for nurses or doctors.

Pharmacists were more likely than non-medical professionals to perceive that pharmacists have this communication role, except for questionnaire items “communicating the effect of the prescribed medication to doctors/nurses,” “communicating the latest information on medicines to nurses,” “participation in conferences regarding surgery,” and “holding education sessions for nurses on drug characteristics.” Non-health care professionals were more likely than pharmacists to perceive that pharmacists should be involved in “holding education sessions for doctors on drug characteristics” (OR 1.40, 95% CI 1.12–1.73, *p* = 0.003).

We observed differences in the opinions of nurses for the questionnaire items “collaboration with nurses on medication administration and management” (OR 0.56, 95% CI 0.34–0.95, *p* = 0.031), “sharing information on the patient’s medication from all attending hospitals with the doctor/nurse” (doctors: OR 0.13, 95% CI 0.05–0.34, *p* < 0.001; nurses: OR 0.60, 95% CI 0.37–0.97, *p* = 0.038), “communicating side effects of prescribed medication to the doctor” (OR 0.66, 95% CI 0.44–1.00, *p* = 0.048), “communicating the patient’s health supplement intake to the doctor” (OR 0.45, 95% CI 0.29–0.71, *p* < 0.001), “providing consultation regarding medication and prescription for the doctor/nurse” (doctors: OR 0.18, 95% CI 0.06–0.51, *p* = 0.001; nurses: OR 0.26, 95% CI 0.10–0.67, *p* = 0.005), “holding education sessions for doctors/nurses on drug characteristics” (doctors: OR 1.66, 95% CI 1.18–2.34, *p* = 0.004; nurses: OR 1.50, 95% CI 1.01–2.23, *p* = 0.045), and “evaluating and suggesting the type and dosage form of medication based on the patient’s condition” (OR 0.38, 95% CI 0.21–0.70, *p* = 0.002). Although pharmacists were more likely than nurses to perceive that they should communicate about most of the above items, nurses were more likely than pharmacists to perceive that pharmacists should be involved in “holding education sessions for doctors/nurses on drug characteristics” (doctors: OR 1.66, 95% CI 1.18–2.34, *p* = 0.004; nurses: OR 1.50, 95% CI 1.01–2.23, *p* = 0.045).

As for medical doctors, there were differences for the questionnaire item “collaboration with doctors on drug treatment plans” (OR 0.50, 95% CI 0.32–0.78, *p* = 0.002), “collaboration with nurses on medication administration and management” (OR 0.40, 95% CI 0.24–0.68, *p* = 0.001), “sharing information on the patient’s medication from all attending hospitals with the doctor/nurse” (doctors: OR 0.19, 95% CI 0.07–0.50, *p* = 0.001; nurses: OR 0.54, 95% CI 0.33–0.88, *p* = 0.014), “communicating the patient’s medication preferences with the doctor” (OR 0.47, 95% CI 0.27–0.83, *p* = 0.009), “communicating the patient’s health supplement intake with the doctor” (OR 0.52, 95% CI 0.33–0.81, *p* = 0.004), “providing consultation regarding medication and prescription for the doctor/nurse” (doctors: OR 0.31, 95% CI 0.11–0.87, *p* = 0.026; nurses: OR 0.22, 95% CI 0.09–0.54, *p* = 0.001), “ communicating the latest information on medicines to the doctor/nurse” (doctors: OR 0.57, 95% CI 0.38–0.83, *p* = 0.004; nurses: OR 0.67, 95% CI 0.49–0.93, *p* = 0.017), and “evaluating and suggesting the type and dosage form of medication based on the patient’s condition” (OR 0.41, 95% CI 0.22–0.75, *p* = 0.004). Pharmacists perceived that pharmacists should communicate with medical doctors or nurses about these items.

### Category 4: Opinions regarding the engagement of pharmacists in community health care (Figs. 7 and 8, Supplementary Table S4)

**Figure 7:**
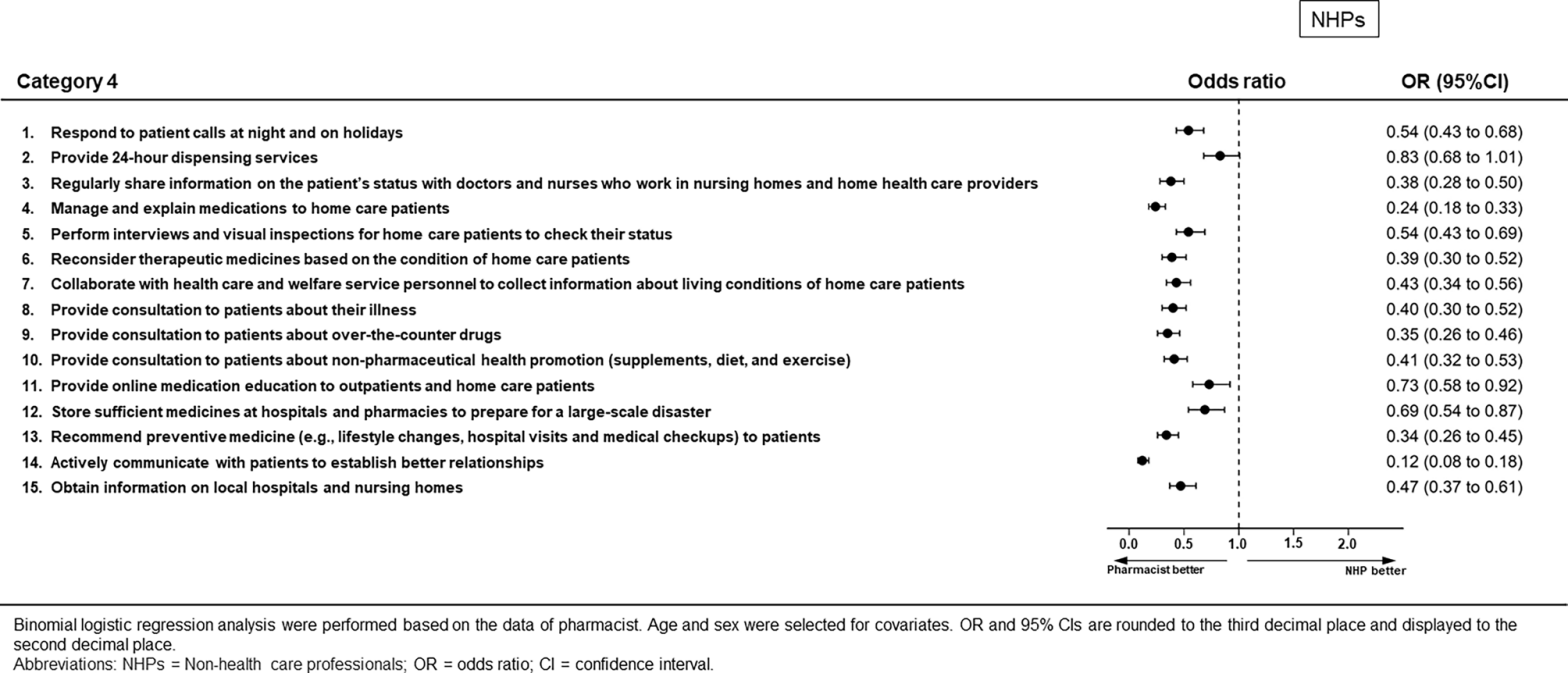
Results of binomial logistic regression analysis for Category 4 for non-health care professionals.

**Figure 8:**
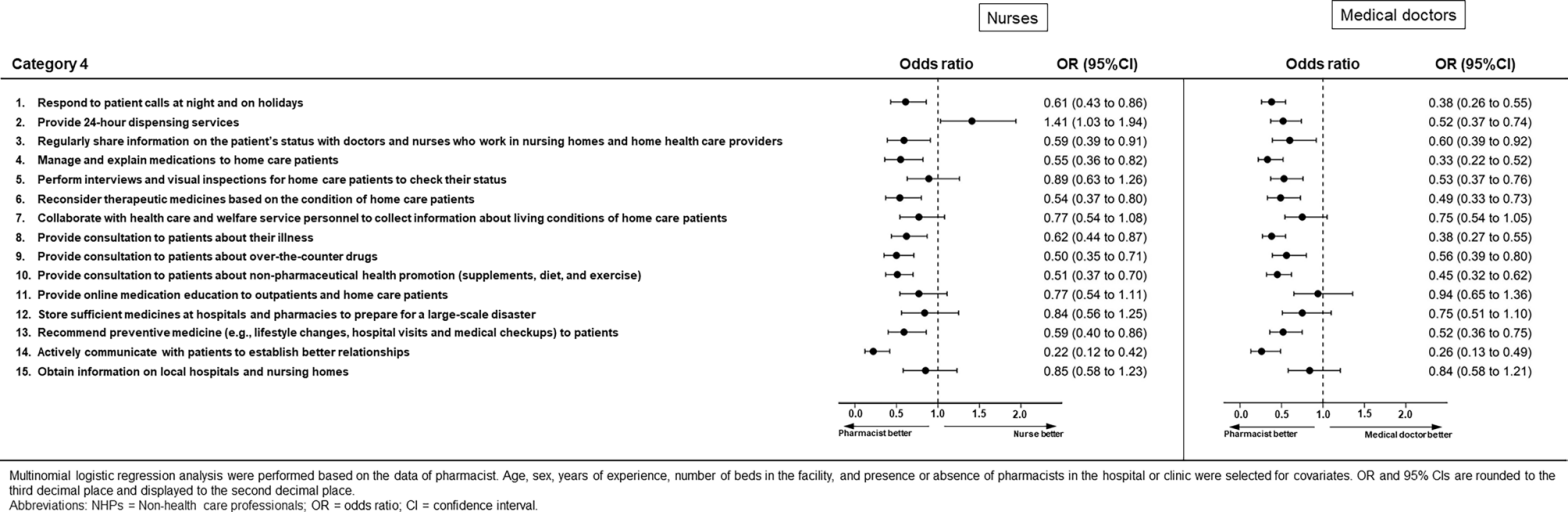
Results of multinomial logistic regression analysis for Category 4 for nurses or doctors.

Pharmacists perceived that they should be engaged in community health care, in comparison with the other three groups of survey respondents. Nurses were more likely than pharmacists to perceive that pharmacists should be engaged in community health care for the questionnaire item “24-h dispensing services” (OR 1.41, 95% CI 1.03–1.94, *p* = 0.035). There were no differences for some questionnaire items between pharmacists and non-health care professionals (“providing 24-h dispensing services”), nurses (“performing interviews and visual inspection for home care patients to check their status,” “collaboration with health care and welfare service personnel to collect information regarding living conditions of home care patients,” “providing online medication education for outpatients and home care patients,” “storing sufficient medicines at hospitals and pharmacies to prepare for a large-scale disaster,” and “obtaining information on local hospitals and nursing homes”), and medical doctors (“collaboration with health care and welfare service personnel to collect information regarding living conditions of home care patients,” “providing online medication education to outpatients and home care patients,” “storing sufficient medicines at hospitals and pharmacies to prepare for a large-scale disaster,” and “obtaining information on local hospitals and nursing homes”).

## Discussion

In this study, we surveyed the opinions of non-medical professionals, pharmacists, nurses, and medical doctors on the roles of pharmacists based on four categories. Each result was compared with responses from pharmacists. Categories 1 and 2 addressed opinions regarding pharmacists’ role in collecting patient information and providing drug information to patients. These tasks are usually considered the basic work of pharmacists^34–36^. These items have been examined in previous studies^27^. However, in our study, we analyzed the data in more detail by developing more specific questions regarding patients’ information. In addition, multiple-choice questions were introduced rather than yes/no responses as used in a previous questionnaire survey conducted in Japan in 2009^27^. Category 3 queried opinions regarding pharmacists’ role in collaboration with health care professionals. Such collaboration is important to effectively use the information obtained in Categories 1 and 2, which leads to the provision of high-quality health care. Category 4 addressed opinions regarding the role of pharmacists in community health care. Pharmacists in Japan are presently less involved in the tasks comprising this category than those in other countries^5^, but their role is expected to be further developed in the future. Questionnaire items regarding Categories 3 and 4 were newly included in our study because of the recently increasing importance of these within the pharmacist profession^37^.

In a comparison between non-health care professionals and pharmacists, pharmacists perceived that they had a greater role in all categories. In this regard, there were no differences between these groups for some items, mainly those directly related to “medicines” (e.g., information on generic drugs, sharing information on the effects of prescribed medication with the doctor or nurse). Additionally, non-health care professionals felt that pharmacists had an important role in holding education sessions for doctors on drug characteristics. These results were similar to those of previous reports and reflect the fact that non-health care professionals perceive pharmacists as “drug experts” rather than experts on health and illness^27,38^. However, non-health care professionals lack understanding regarding the basic work of pharmacists, such as collecting and providing information about side effects, medication status, and drug interactions^34–36^ (Categories 1 and 2). In other words, recognition of pharmacists as drug experts is limited among non-health care professionals. Pharmacists in Japan tend to place greater emphasis on dispensing work rather than on interpersonal work; this may be a reason for non-health care professionals contacting medical doctors for a problem rather than pharmacists. Therefore, non-health care professionals may have little understanding about the recent development and diversity of pharmaceutical services^39^. “Patient-centered medicine,” in which patients themselves are actively involved in decision-making together with medical professionals, has been promoted in recent years^40,41^. Additionally, self-medication and community health care (Category 4) are becoming increasingly important. The Japan Pharmaceutical Association states that the role of the pharmacist is to help consumers use medicines appropriately and avoid adverse events, with several examples presented^42^. For patients to receive appropriate medical care, appropriate medical information and techniques are required. To advance these concepts, the cooperation of medical professionals within their own specialization is understandably key. However, if non-health care professionals do not understand the role of the pharmacist, they will not rely on these professionals, which can affect cooperation and patient care. Therefore, pharmacists must help to expand perceptions among non-health care professionals regarding the role and work of pharmacists. In Canada, efforts have been made to help the general public understand the work of pharmacists by disseminating information about their activities through videos and social networking sites^43,44^. Providing opportunities to actively share information in this way is important for pharmacists to contribute to health care. During the COVID-19 pandemic, the tendency for people to avoid contact with others has led to social isolation^45,46^. In a socially distanced society, communication between non-health care professionals and pharmacists is extremely important, for instance, in following up with patients after prescribing medications^47^. In this way, non-health care professionals can better understand the roles of pharmacists and come to rely on them. Moreover, trusting relationships facilitate the participation of various medical professionals and may improve the quality of medical care received by patients^20–23^.

Although nurses and medical doctors perceived smaller roles for pharmacists in Categories 1 and 2, differences between the perceptions of pharmacists themselves were smaller than the differences with non-health care professionals. This result suggests that nurses and medical doctors in our study understood the basic role of the pharmacist. However, they had little knowledge that pharmacists can collect medical data and inform patients about forgetting to take their medication. Moreover, nurses had little understanding about the role of pharmacists in collecting information regarding patients’ attendance at medical institutions. Because nurses and medical doctors perceive their own role in this regard in daily medical care, they may not believe that this is a role for pharmacists. In contrast, nurses but not medical doctors were aware that pharmacists provide patients with information on generic drugs and medical facilities such as local hospitals, pharmacies, and nursing care facilities. Because medical doctors prescribe medicines and write referrals for patients, they may not consider that pharmacists can also provide this information to patients. Nurses do not have as many opportunities as medical doctors to provide such information. However, they have more opportunities to interact with patients than pharmacists or medical doctors and need to respond to various requests or questions from the patient. Therefore, nurses may feel that pharmacists should provide patients with information on generic drugs and medical facilities.

With respect to communication with medical staff (Category 3), opinions among the three groups were generally consistent in terms of communicating information regarding drug effects, side effects, and how to take medications. However, because medical doctors prescribe drugs for patients and are the main provider of medical care, nurses and medical doctors are less aware about the role of pharmacists in communicating information to them regarding drug treatment plans and patients’ medication preferences, consulting with regard to medication and prescription, and suggesting the type and dosage form of medication. It is difficult to fill these gaps in opinion, but pharmacists can inform medical staff as well as non-health care professionals to help them understand the many roles played by a pharmacist. For instance, the prescription refill system, which began in 2022 in Japan, requires that pharmacists carefully confirm the physical condition of a patient and promptly share the information with medical doctors if any irregularities are found^48,49^. In this regard, to disseminate this new role, pharmacists need to improve their capabilities in clinical judgment^50,51^. In doing so, better cooperation and collaboration among specialists is possible, which can lead to better patient-centered health care^20–23^.

In other questionnaire items, nurses but not medical doctors perceived that pharmacists are involved in holding education sessions for doctors on drug characteristics. This result indicated that nurses as well as non-health care professionals recognize pharmacists as drug experts. In contrast, because medical doctors must study the characteristics of drug for themselves to prescribe medicines, receiving educational sessions from pharmacists may not seem necessary to physicians.

The largest difference in opinion between nurses or medical doctors and pharmacists was regarding the involvement of pharmacists in community health care (Category 4). The perceptions of the former two groups were poorer than those of pharmacists. In the case of community health care, each medical professional should make effort to break down barriers regarding their specialty and create overlapping areas to provide better medical care. In Japan, the “family pharmacist system” was introduced in 2016^52^. This system is intended to identify and manage the medicines used concomitantly by patients, including over-the-counter medicines. Additionally, pharmacists provide comprehensive health guidance and support patients’ needs by offering home visits or telephone consultations. However, these tasks have mainly been carried out by medical doctors in the past. Therefore, it is possible that they are reluctant to accept medical roles for pharmacists. For example, the introduction of “pharmacist prescribing,” which is attracting attention as a new job function among pharmacists, is reported to have met with resistance from medical professionals in some countries^53^. The activities of pharmacists during the COVID-19 pandemic have been underestimated; however, various health care-related activities were performed by pharmacists worldwide during this period, such as providing the general public with information about infection control, developing drug supply systems, and responding to public health issues^43^. Therefore, as a first step in cooperation, medical professionals should actively improve mutual understanding of their respective professions^54,55^. Pharmacists should take the initiative to communicate with other medical staff to help them better understand the current roles of pharmacists.

Our research has some limitations. First, our study was performed in medium-sized cities of Japan. Because the role of the pharmacist depends on the local environment, the opinion of pharmacists’ work may differ between rural and urban areas. Second, complete data on the backgrounds of non-health care professionals could not be collected. Questionnaire responses from non-health care professionals may have varied depending on whether they had been in contact with a pharmacist in a pharmacy or hospital and whether they had experience with home visits. From another perspective, the economic situation or family structure can affect the experience of visiting a hospital. Although it was necessary to consider various backgrounds in the case of non-health care professionals, this information was not collected owing to difficulty with the questionnaire format and its incorporation into the analysis. In the future, it will be necessary to verify the results by taking these factors into account. Third, because we limited the number of questionnaire items to avoid inappropriate answers, other important items regarding the opinion of pharmacists’ roles may have been missed. Fourth, because the survey among non-health care professionals was conducted via a web-based questionnaire administered by registered monitors, respondents needed to have access to the Internet. Therefore, because the responses of those who do not use the Internet are not included, there is a possibility that actual perceptions may differ, especially among older people, a group in whom the difference between the number of Internet users and non-users is considered to be large. Although our survey has some limitations and further detailed investigation is needed, the results of this study can contribute to the provision of high-quality patient health care in Japan.

## Conclusion

In this study, we found that perceptions of non-health care professionals regarding the scope of pharmacists’ work were poorer than those of pharmacists. However, opinions were similar between pharmacists, nurses, and medical doctors regarding the basic work of pharmacists. Perceptions related to new pharmacist roles, such as in health care collaboration and community health care, were low among nurses and medical doctors, as compared with pharmacists. For pharmacists to contribute in health care, non-health care professionals and health care professionals must be properly informed about the pharmacy specialty and the role of pharmacists in health care.

## Supplementary Information

Supplementary Information.

## Data Availability

The data supporting the findings of this study are available on request from T. Ikeda. The data are not publicly available as they contain information that could compromise the privacy of research participants.
