# Supplementary material for: Questionnaire survey on pharmacists’ roles among non- and health care professionals in medium-sized cities in Japan
Source: Sci Rep. 2023 Apr 4;13:5458. doi: 10.1038/s41598-023-32777-0 (PMC10071258; doi:10.1038/s41598-023-32777-0)
Supplement: Supplementary file 1 — Supplementary Information. [file 41598_2023_32777_MOESM1_ESM.pdf]

Supplementary Information

Questionnaire survey on pharmacists' roles among non- and health care professionals in medium-sized cities in Japan

Fukuko Horio[1*], Tokunori Ikeda[1*], Yanosuke Kouzaki[2], Tomoo Hirahara[3], Kengo Masa[4], Sawana Narita[4], Yusuke Tomita[5], Shu Tsuruzoe[6], Akihiko Fujisawa[7], Yuki Akinaga[1], Yoko Ashizuka[1], Yuki Inoue[1], Ayaka Unten[1], Katsutoshi Okamura[1], Yuiko Takechi[1], Yasuhiro Takenouchi[1], Fuka Tanaka[1], Chiharu Masuda[1], Yusuke Sugimura[8], Yuji Uchida[1]

Author affiliations

[1]Laboratory of Clinical Pharmacology and Therapeutics, Faculty of Pharmaceutical Sciences, Sojo University, Kumamoto, Japan

[2]Department of Neurology, National Hospital Organization Kumamoto Medical Center, Kumamoto, Japan

[3]Department of Neurology, Uki General Hospital, Kumamoto, Japan

[4]Department of Pharmacy, Kumamoto University Hospital, Kumamoto, Japan

[5]Department of Respiratory Medicine, Graduate School of Medical Sciences, Kumamoto University, Kumamoto, Japan

[6]Musashigaoka Hospital, Kumamoto, Japan

[7]Kumamoto Dermatology, Plastic Surgery Clinic, Kumamoto, Japan

[8]Department of Neurology, Sugimura Hospital, Kumamoto, Japan

***Corresponding authors**

Fukuko Horio, Laboratory of Clinical Pharmacology and Therapeutics, Faculty of Pharmaceutical Sciences, Sojo University, 4-22-1, Ikeda, Kumamoto 860-0082, Japan. Phone: +81-96-326-3111, Fax: +81-96-326-3000, fhorio@ph.sojo-u.ac.jp

Tokunori Ikeda, Laboratory of Clinical Pharmacology and Therapeutics, Faculty of Pharmaceutical Sciences, Sojo University, 4-22-1, Ikeda, Kumamoto 860-0082, Japan. Phone: +81-96-326-3111, Fax: +81-96-326-3000, ryousei@ph.sojo-u.ac.jp

Supplementary Figure S1. Map of Japan and Kyushu region.

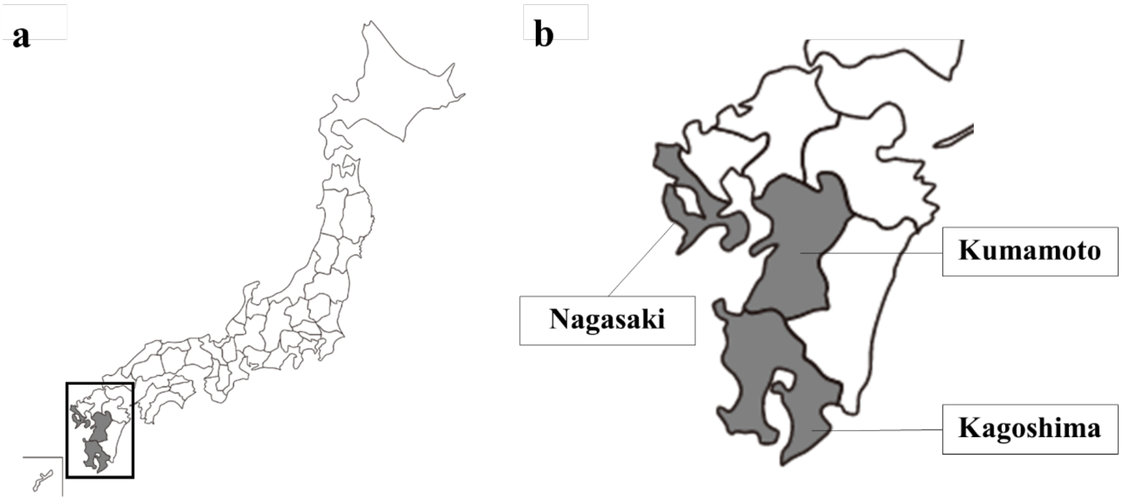

(a) Map of Japan. The area in the square is the Kyushu region. The gray solid areas show Kumamoto, Kagoshima, and Nagasaki prefectures.

(b) Map of the Kyushu region (in the square in Figure S1a). The gray solid areas show Kumamoto, Kagoshima, and Nagasaki prefectures. Non-health care professionals were surveyed throughout the Kyushu region. Health care professionals were surveyed in Kumamoto, Kagoshima, Nagasaki prefectures. We used a web-based map tool (https://www.freemap.jp/free.html) to create the figures.

Supplementary Table S1. Survey responses for Category 1.

Category 1: Do you think that pharmacists should collect patients' information regarding the following items?

(Scores: 1 = completely disagree, 2 = somewhat disagree, 3= unsure, 4 = somewhat agree, 5 = completely agree)

	**Pharmacists**	**Non-health care professionals**	**Nurses**	**Medical doctors**	***p*-value**		
	n = 204	n = 487	n = 151	n = 133	**Pharmacists vs. Non-health care professionals**	**Pharmacists vs. Nurses**	**Pharmacists vs. Medical doctors**
**Obtaining basic patient information**							
**1. Family structure**	2 (1.0)	56 (11.5)	2 (1.3)	2 (1.5)	NA	< 0.001	0.16
	5 (2.5)	81 (16.6)	10 (6.7)	10 (7.6)			
	16 (7.9)	134 (27.5)	30 (20.0)	9 (6.8)			
	136 (67.0)	162 (33.3)	62 (41.3)	72 (54.5)			
	44 (21.7)	54 (11.1)	46 (30.7)	39 (29.5)			
**2. Eating habits**	1 (0.5)	13 (2.7)	0 (0.0)	3 (2.3)	< 0.001	0.007	0.07
	2 (1.0)	27 (5.5)	7 (4.7)	6 (4.5)			
	5 (2.5)	74 (15.2)	15 (10.0)	9 (6.8)			
	111 (54.7)	232 (47.6)	77 (51.3)	67 (50.8)			
	84 (41.4)	141 (29.0)	51 (34.0)	47 (35.6)			
**3. Attending medical institutions**	0 (0.0)	12 (2.5)	1 (0.7)	0 (0.0)	< 0.001	< 0.001	0.005
	1 (0.5)	11 (2.3)	1 (0.7)	3 (2.3)			
	5 (2.5)	45 (9.2)	8 (5.3)	2 (1.5)			
	26 (12.7)	200 (41.1)	51 (34.0)	36 (27.3)			

	172 (84.3)	219 (45.0)	89 (59.3)	91 (68.9)			
**4. Household income**	7 (3.4)	124 (25.5)	9 (6.0)	8 (6.1)	NA	1.00	0.30
	24 (11.8)	133 (27.3)	18 (12.0)	12 (9.1)			
	98 (48.0)	160 (32.9)	63 (42.0)	48 (36.4)			
	67 (32.8)	58 (11.9)	48 (32.0)	54 (40.9)			
	8 (3.9)	12 (2.5)	12 (8.0)	10 (7.6)			
**5. Job description**	3 (1.5)	56 (11.5)	2 (1.3)	2 (1.5)	NA	0.005	0.19
	5 (2.5)	86 (17.7)	11 (7.3)	12 (9.1)			
	28 (13.8)	130 (26.7)	31 (20.7)	21 (15.9)			
	137 (67.5)	184 (37.8)	71 (47.3)	75 (56.8)			
	30 (14.8)	31 (6.4)	35 (23.3)	22 (16.7)			
**Patient's disease status and medication administration**							
**1. Present illness**	0 (0.0)	3 (0.6)	0 (0.0)	0 (0.0)	< 0.001	0.70	0.54
	0 (0.0)	4 (0.8)	2 (1.3)	0 (0.0)			
	0 (0.0)	36 (7.4)	1 (0.7)	0 (0.0)			
	15 (7.4)	160 (32.9)	12 (7.9)	16 (12.0)			
	187 (92.6)	284 (58.3)	136 (90.1)	117 (88.0)			
**2. Past medical history**	0 (0.0)	5 (1.0)	0 (0.0)	0 (0.0)	< 0.001	0.83	0.77
	0 (0.0)	8 (1.6)	2 (1.3)	1 (0.8)			
	2 (1.0)	39 (8.0)	3 (2.0)	2 (1.5)			
	34 (16.7)	208 (42.7)	20 (13.2)	30 (22.6)			
	167 (82.3)	227 (46.6)	126 (83.4)	100 (75.2)			

**3. Medical data such as blood sampling results**	0 (0.0)	9 (1.8)	1 (0.7)	0 (0.0)	< 0.001	0.002	< 0.001
	0 (0.0)	9 (1.8)	1 (0.7)	0 (0.0)			
	0 (0.0)	102 (20.9)	9 (6.0)	8 (6.0)			
	46 (22.7)	196 (40.2)	32 (21.2)	54 (40.6)			
	157 (77.3)	171 (35.1)	108 (71.5)	71 (53.4)			
**4. Medicine taken and medication interactions**	0 (0.0)	2 (0.4)	0 (0.0)	0 (0.0)	< 0.001	0.039	0.022
	0 (0.0)	7 (1.4)	0 (0.0)	0 (0.0)			
	0 (0.0)	39 (8.0)	1 (0.7)	0 (0.0)			
	3 (1.5)	128 (26.3)	10 (6.6)	10 (7.5)			
	200 (98.5)	311 (63.9)	140 (92.7)	123 (92.5)			
**5. History of medication**	0 (0.0)	3 (0.6)	1 (0.7)	0 (0.0)	< 0.001	0.048	0.92
	0 (0.0)	11 (2.3)	2 (1.3)	1 (0.8)			
	5 (2.5)	61 (12.5)	3 (2.0)	6 (4.5)			
	84 (41.4)	169 (34.7)	42 (28.0)	47 (35.3)			
	114 (56.2)	243 (49.9)	102 (68.0)	79 (59.4)			
**6. Side effect history**	0 (0.0)	3 (0.6)	0 (0.0)	0 (0.0)	< 0.001	0.016	< 0.001
	0 (0.0)	8 (1.6)	0 (0.0)	0 (0.0)			
	0 (0.0)	39 (8.0)	0 (0.0)	0 (0.0)			
	1 (0.5)	118 (24.2)	8 (5.3)	14 (10.5)			
	202 (99.5)	319 (65.5)	142 (94.7)	119 (89.5)			
**7. Confirmation of how much medication the patient forgot to take and the amount**	0 (0.0)	2 (0.4)	0 (0.0)	0 (0.0)	< 0.001	1.00	0.21
	0 (0.0)	13 (2.7)	0 (0.0)	1 (0.8)			
	1 (0.5)	67 (13.8)	2 (1.3)	5 (3.8)			

**of medication**	41 (20.2)	209 (42.9)	37 (24.5)	26 (19.5)			
**remaining**	161 (79.3)	196 0.2)	112 (74.2)	101 (75.9)			
**8. Patient's concerns and wishes regarding medication (cost of medication, type of medication, increase or decrease in dosage)**	0 (0.0)	1 (0.2)	0 (0.0)	0 (0.0)	< 0.001	1.00	1.00
	0 (0.0)	10 (2.1)	0 (0.0)	0 (0.0)			
	2 (1.0)	65 (13.3)	2 (1.3)	4 (3.0)			
	54 (26.7)	196 (40.2)	33 (21.9)	34 (25.6)			
	146 (72.3)	215 (44.1)	116 (76.8)	95 (71.4)			

Supplementary Table S2. Survey responses for Category 2.

Category 2: Do you think pharmacists should provide information regarding the following items to patients?

(Scores: 1 = completely disagree, 2 = somewhat disagree, 3= unsure, 4 = somewhat agree, 5 = completely agree)

	Pharmacists	Non-health care professionals	Nurses	Medical doctors	*p*-value		
	n = 204	n = 487	n = 151	n = 133	Pharmacists vs. Non-health care professionals	Pharmacists vs. Nurses	Pharmacists vs. Medical doctors
**1. Safety information on medicines**	0 (0.0)	3 (0.6)	0 (0.0)	0 (0.0)	0.026	0.93	0.13
	1 (0.5)	20 (4.1)	2 (1.3)	0 (0.0)			
	15 (7.4)	52 (10.7)	9 (6.0)	3 (2.3)			
	73 (36.1)	192 (39.4)	43 (28.5)	40 (30.1)			
	113 (55.9)	220 (45.2)	97 (64.2)	90 (67.7)			
**2. Information on generic drugs**	0 (0.0)	4 (0.8)	0 (0.0)	0 (0.0)	0.65	1.00	1.00
	4 (2.0)	9 (1.8)	2 (1.3)	4 (3.0)			
	30 (14.9)	67 (13.8)	23 (15.2)	14 (10.5)			
	97 (48.0)	197 (40.5)	62 (41.1)	66 (49.6)			
	71 (35.1)	210 (43.1)	64 (42.4)	49 (36.8)			
**3. Education on correspondence in the case of forgetting to take medication**	1 (0.5)	5 (1.0)	0 (0.0)	0 (0.0)	< 0.001	0.07	0.73
	0 (0.0)	7 (1.4)	1 (0.7)	2 (1.5)			
	1 (0.5)	62 (12.7)	5 (3.3)	2 (1.5)			
	57 (28.2)	207 (42.5)	29 (19.2)	42 (31.6)			
	143 (70.8)	206 (42.3)	116 (76.8)	87 (65.4)			

**4. Education on correspondence in the case of drug-induced side effects**	0 (0.0)	6 (1.2)	0 (0.0)	0 (0.0)	< 0.001	1.00	1.00
	1 (0.5)	2 (0.4)	0 (0.0)	1 (0.8)			
	4 (2.0)	49 (10.1)	4 (2.6)	1 (0.8)			
	48 (23.8)	158 (32.4)	26 (17.2)	32 (24.1)			
	149 (73.8)	272 (55.9)	121 (80.1)	99 (74.4)			
**5. Information on local hospitals, pharmacies, and nursing care facilities**	2 (1.0)	8 (1.6)	4 (2.6)	4 (3.0)	0.043	0.003	0.62
	17 (8.4)	25 (5.1)	13 (8.6)	9 (6.8)			
	63 (31.2)	120 (24.6)	31 (20.5)	32 (24.1)			
	88 (43.6)	206 (42.3)	53 (35.1)	57 (42.9)			
	32 (15.8)	128 (26.3)	50 (33.1)	31 (23.3)			

Supplementary Table S3. Survey responses for Category 3.

Category 3: Do you think pharmacists should communicate with medical doctors or nurses in the following ways?

(Scores: 1 = completely disagree, 2 = somewhat disagree, 3= unsure, 4 = somewhat agree, 5 = completely agree)

	**Pharmacists**	**Non-health care professionals**	**Nurses**	**Medical doctors**	***p*-value**		
	n = 204	n = 487	n = 151	n = 133	**Pharmacists vs. Non-health care professionals**	**Pharmacists vs. Nurses**	**Pharmacists vs. Medical doctors**
**1. Collaborate with doctors on drug treatment plans**	0 (0.0)	8 (1.6)	1 (0.7)	1 (0.8)	0.011	1.00	1.00
	2 (1.0)	15 (3.1)	2 (1.3)	4 (3.0)			
	29 (14.3)	106 (21.8)	19 (12.6)	14 (10.5)			
	87 (42.9)	209 (42.9)	63 (41.7)	60 (45.1)			
	85(41.9)	149 (30.6)	66 (43.7)	54 (40.6)			
**2. Collaborate with nurses on medication administration and management**	1 (0.5)	7 (1.4)	0 (0.0)	0 (0.0)	< 0.001	1.00	1.00
	1 (0.5)	27 (5.5)	2 (1.3)	3 (2.3)			
	15 (7.5)	139 (28.5)	8 (5.3)	10 (7.5)			
	97 (48.3)	200 (41.1)	70 (46.4)	64 (48.1)			
	87 (43.3)	114 (23.4)	71 (47.0)	56 (42.1)			
**3. Share information with the doctor on the patient's medication from all attending hospitals**	0 (0.0)	4 (0.8)	0 (0.0)	0 (0.0)	< 0.001	0.19	0.81
	0 (0.0)	7 (1.4)	2 (1.3)	0 (0.0)			
	4 (2.0)	78 (16.0)	3 (2.0)	3 (2.3)			
	29 (14.4)	203 (41.7)	34 (22.5)	28 (21.1)			

	169 (83.7)	195 (40.0)	112 (74.2)	102 (76.7)			
**4. Share information with the nurse on the patient's medication from all attending hospitals**	1 (0.5)	5 (1.0)	0 (0.0)	0 (0.0)	< 0.001	0.73	1.00
	2 (1.0)	18 (3.7)	2 (1.3)	2 (1.5)			
	21 (10.4)	110 (22.6)	8 (5.3)	10 (7.5)			
	75 (37.1)	201 (41.3)	51 (33.8)	49 (36.8)			
	103 (51.0)	153 (31.4)	90 (59.6)	72 (54.1)			
**5. Share information with the doctor on the effects of prescribed medication**	6 (3.0)	8 (1.6)	1 (0.7)	2 (1.5)	0.60	0.20	0.87
	12 (5.9)	14 (2.9)	7 (4.6)	6 (4.5)			
	42 (20.7)	98 (20.1)	21 (13.9)	18 (13.5)			
	78 (38.4)	185 (38.0)	54 (35.8)	64 (48.1)			
	65 (32.0)	182 (37.4)	68 (45.0)	43 (32.3)			
**6. Share information with the nurse on the effects of prescribed medication**	5 (2.5)	8 (1.6)	1 (0.7)	1 (0.8)	1.00	0.003	1.00
	6 (3.0)	21 (4.3)	5 (3.3)	4 (3.0)			
	42 (20.7)	121 (24.8)	14 (9.3)	21 (15.8)			
	93 (45.8)	201 (41.3)	61 (40.4)	66 (49.6)			
	57 (28.1)	136 (27.9)	70 (46.4)	41 (30.8)			
**7. Share information with the doctor on side effects of prescribed medication**	0 (0.0)	7 (1.4)	0 (0.0)	2 (1.5)	0.006	0.68	0.042
	4 (2.0)	9 (1.8)	6 (4.0)	2 (1.5)			
	17 (8.4)	75 (15.4)	20 (13.2)	11 (8.3)			
	66 (32.5)	189 (38.8)	51 (33.8)	63 (47.4)			
	116 (57.1)	207 (42.5)	74 (49.0)	55 (41.4)			

**8. Share information with the nurse on side effects of prescribed medication**	2 (1.0)	6 (1.2)	1 (0.7)	1 (0.8)	0.016	1.00	1.00
	3 (1.5)	13 (2.7)	5 (3.3)	2 (1.5)			
	25 (12.3)	104 (21.4)	12 (7.9)	11 (8.3)			
	80 (39.4)	205 (42.1)	54 (35.8)	65 (48.9)			
	93 (45.8)	159 (32.6)	79 (52.3)	54 (40.6)			
**9. Share information with the doctor on how the patient takes their medicine**	0 (0.0)	4 (0.8)	0 (0.0)	0 (0.0)	< 0.001	0.013	1.00
	0 (0.0)	17 (3.5)	3 (2.0)	1 (0.8)			
	4 (2.0)	75 (15.4)	13 (8.6)	2 (1.5)			
	80 (39.4)	201 (41.3)	55 (36.4)	51 (38.3)			
	119 (58.6)	190 (39.0)	80 (53.0)	79 (59.4)			
**10. Share information with the nurse on how the patient takes their medicine**	2 (1.0)	7 (1.4)	1 (0.7)	0 (0.0)	< 0.001	1.00	1.00
	2 (1.0)	23 (4.7)	3 (2.0)	2 (1.5)			
	18 (8.9)	117 (24.0)	12 (7.9)	8 (6.0)			
	78 (38.4)	200 (41.1)	53 (35.1)	54 (40.6)			
	103 (50.7)	140 (28.7)	82 (54.3)	69 (51.9)			
**11. Share information with the doctor on the patient's medication preferences**	0 (0.0)	3 (0.6)	0 (0.0)	0 (0.0)	< 0.001	0.99	1.00
	1 (0.5)	14 (2.9)	1 (0.7)	1 (0.8)			
	2 (1.0)	87 (17.9)	4 (2.6)	3 (2.3)			
	67 (33.2)	194 (39.8)	40 (26.5)	53 (39.8)			
	132 (65.3)	189 (38.8)	106 (70.2)	76 (57.1)			
**12. Share information with the nurse on the patient's medication preferences**	2 (1.0)	9 (1.8)	1 (0.7)	0 (0.0)	0.002	< 0.001	0.09
	4 (2.0)	18 (3.7)	5 (3.3)	6 (4.5)			
	36 (17.7)	127 (26.1)	6 (4.0)	12 (9.0)			

	70 (34.5)	197 (40.5)	48 (32.0)	61 (45.9)			
	91 (44.8)	136 (27.9)	90 (60.0)	54 (40.6)			
**13. Share information with the doctor on the patient's health supplement intake**	0 (0.0)	7 (1.4)	1 (0.7)	0 (0.0)	< 0.001	0.47	1.00
	3 (1.5)	40 (8.2)	5 (3.3)	3 (2.3)			
	21 (10.3)	120 (24.6)	25 (16.6)	16 (12.0)			
	81 (39.9)	172 (35.3)	51 (33.8)	59 (44.4)			
	98 (48.3)	148 (30.4)	69 (45.7)	55 (41.4)			
**14. Share information with the nurse on the patient's health supplement intake**	2 (1.0)	13 (2.7)	1 (0.7)	0 (0.0)	0.17	1.00	0.64
	10 (4.9)	38 (7.8)	6 (4.0)	4 (3.0)			
	47 (23.2)	146 (30.0)	27 (17.9)	22 (16.5)			
	84 (41.4)	178 (36.6)	63 (41.7)	70 (52.6)			
	60 (29.6)	112 (23.0)	54 (35.8)	37 (27.8)			
**15. Provide consultation regarding medication and prescription to the doctor**	0 (0.0)	2 (0.4)	0 (0.0)	0 (0.0)	< 0.001	0.002	0.022
	0 (0.0)	8 (1.6)	0 (0.0)	0 (0.0)			
	0 (0.0)	78 (16.0)	4 (2.6)	4 (3.0)			
	15 (7.4)	197 (40.5)	26 (17.2)	17 (12.8)			
	188 (92.6)	202 (41.5)	121 (80.1)	112 (84.2)			
**16. Provide consultation regarding medication and prescription to the nurse**	0 (0.0)	5 (1.0)	0 (0.0)	1 (0.8)	< 0.001	1.00	< 0.001
	0 (0.0)	14 (2.9)	0 (0.0)	0 (0.0)			
	2 (1.0)	108 (22.2)	3 (2.0)	7 (5.3)			
	23 (11.3)	195 (40.0)	23 (15.2)	34 (25.6)			
	178 (87.7)	165 (33.9)	125 (82.8)	91 (68.4)			

**17. Provide the latest information on medicines to the doctor**	3 (1.5)	4 (0.8)	0 (0.0)	2 (1.5)	0.15	0.62	1.00
	5 (2.5)	15 (3.1)	2 (1.3)	6 (4.5)			
	24 (11.8)	85 (17.5)	21 (13.9)	18 (13.5)			
	69 (34.0)	193 (39.6)	39 (25.8)	54 (40.6)			
	102 (50.2)	190 (39.0)	89 (58.9)	53 (39.8)			
**18. Provide the latest information on medicines to the nurse**	4 (2.0)	6 (1.2)	2 (1.3)	3 (2.3)	1.00	0.72	1.00
	10 (4.9)	18 (3.7)	11 (7.3)	10 (7.5)			
	55 (27.1)	134 (27.5)	29 (19.2)	32 (24.1)			
	69 (34.0)	185 (38.0)	47 (31.1)	52 (39.1)			
	65 (32.0)	144 (29.6)	62 (41.1)	36 (27.1)			
**19. Participate in conferences regarding discharge of hospitalized patients**	3 (1.5)	13 (2.7)	1 (0.7)	0 (0.0)	NA	0.09	0.44
	7 (3.5)	36 (7.4)	7 (4.7)	7 (5.3)			
	25 (12.4)	174 (35.7)	31 (20.8)	21 (15.9)			
	97 (48.0)	176 (36.1)	49 (32.9)	72 (54.5)			
	70 (34.7)	88 (18.1)	61 (40.9)	32 (24.2)			
**20. Participate in conferences regarding surgery in hospitalized patients**	8 (4.0)	14 (2.9)	3 (2.0)	1 (0.8)	1.00	0.06	1.00
	14 (6.9)	31 (6.4)	6 (4.0)	9 (6.8)			
	75 (37.1)	178 (36.6)	44 (29.5)	47 (35.6)			
	75 (37.1)	178 (36.6)	53 (35.6)	55 (41.7)			
	30 (14.9)	86 (17.7)	43 (28.9)	20 (15.2)			
**21. Hold education sessions for doctors on drug characteristics**	4 (2.0)	4 (0.8)	1 (0.7)	4 (3.0)	< 0.001	< 0.001	0.16
	16 (7.9)	15 (3.1)	1 (0.7)	6 (4.5)			
	73 (36.1)	144 (29.6)	37 (24.8)	36 (27.1)			

	78 (38.6)	181 (37.2)	60 (40.3)	51 (38.3)			
	31 (15.3)	143 (29.4)	50 (33.6)	36 (27.1)			
**22. Hold education sessions for nurses on drug characteristics**	2 (1.0)	3 (0.6)	1 (0.7)	2 (1.5)	0.25	< 0.001	1.00
	10 (5.0)	16 (3.3)	1 (0.7)	8 (6.0)			
	50 (24.8)	148 (30.4)	19 (12.8)	28 (21.1)			
	100 (49.5)	196 (40.2)	64 (43.2)	65 (48.9)			
	40 (19.8)	124 (25.5)	63 (42.6)	30 (22.6)			
**23. Evaluate and suggest the type and dosage form of medication based on the patient's condition (e.g., capsules, syrup)**	1 (0.5)	6 (1.2)	1 (0.7)	0 (0.0)	< 0.001	0.001	0.001
	0 (0.0)	16 (3.3)	0 (0.0)	0 (0.0)			
	1 (0.5)	126 (25.9)	11 (7.4)	5 (3.8)			
	45 (22.3)	211 (43.3)	43 (28.9)	50 (37.6)			
	155 (76.7)	128 (26.3)	94 (63.1)	78 (58.6)			

Supplementary Table S4. Survey responses for Category 4.

Category 4: Do you think pharmacists should be engaged in community health care in the following ways?

(Scores: 1 = completely disagree, 2 = somewhat disagree, 3= unsure, 4 = somewhat agree, 5 = completely agree)

	**Pharmacists**	**Non-health care professionals**	**Nurses**	**Medical doctors**	***p*-value**		
	n = 204	n = 487	n = 151	n = 133	**Pharmacists vs. Non-health care professionals**	**Pharmacists vs. Nurses**	**Pharmacists vs. Medical doctors**
**1. Respond to patient calls at night and on holidays**	5 (2.5)	19 (3.9)	5 (3.4)	9 (6.8)	< 0.001	< 0.001	< 0.001
	6 (3.0)	45 (9.2)	21 (14.1)	26 (19.5)			
	50 (24.8)	170 (34.9)	56 (37.6)	50 (37.6)			
	94 (46.5)	179 (36.8)	49 (32.9)	39 (29.3)			
	47 (23.3)	74 (15.2)	18 (12.1)	9 (6.8)			
**2. Provide 24-hour dispensing services**	8 (4.0)	28 (5.7)	6 (4.0)	15 (11.3)	1.00	1.00	< 0.001
	23 (11.4)	68 (14.0)	15 (10.1)	37 (27.8)			
	87 (43.1)	220 (45.2)	66 (44.3)	52 (39.1)			
	66 (32.7)	123 (25.3)	39 (26.2)	26 (19.5)			
	18 (8.9)	48 (9.9)	23 (15.4)	3 (2.3)			
**3. Regularly share information on the patient's status with doctors and nurses who work in nursing homes**	0 (0.0)	2 (0.4)	0 (0.0)	3 (2.3)	< 0.001	0.005	< 0.001
	2 (1.0)	27 (5.5)	4 (2.7)	3 (2.3)			
	18 (8.9)	153 (31.4)	29 (19.6)	20 (15.0)			
	105 (52.0)	210 (43.1)	80 (54.1)	86 (64.7)			
	77 (38.1)	95 (19.5)	35 (23.6)	21 (15.8)			

**and home health care providers**							
**4. Manage and explain medications to home care patients**	1 (0.5)	8 (1.6)	1 (0.7)	4 (3.1)	NA	< 0.001	< 0.001
	1 (0.5)	39 (8.0)	2 (1.4)	9 (6.9)			
	10 (5.0)	177 (36.3)	42 (28.4)	40 (30.5)			
	84 (41.6)	185 (38.0)	63 (42.6)	63 (48.1)			
	106 (52.5)	78 (16.0)	40 (27.0)	15 (11.5)			
**5. Perform interviews and visual inspections for home care patients to check their status**	2 (1.0)	15 (3.1)	3 (2.0)	5 (3.8)	NA	0.31	< 0.001
	5 (2.5)	44 (9.0)	10 (6.7)	17 (12.8)			
	40 (19.8)	175 (35.9)	39 (26.2)	43 (32.3)			
	103 (51.0)	179 (36.8)	67 (45.0)	56 (42.1)			
	52 (25.7)	74 (15.2)	30 (20.1)	12 (9.0)			
**6. Reconsider therapeutic medicines based on the condition of home care patients**	0 (0.0)	6 (1.2)	2 (1.3)	3 (2.3)	< 0.001	< 0.001	< 0.001
	1 (0.5)	29 (6.0)	4 (2.7)	6 (4.5)			
	25 (12.4)	156 (32.0)	37 (24.8)	26 (19.5)			
	83 (41.1)	185 (38.0)	66 (44.3)	70 (52.6)			
	93 (46.0)	111 (22.8)	40 (26.8)	28 (21.1)			
**7. Collaborate with health care and welfare service personnel to collect information about living conditions**	2 (1.0)	7 (1.4)	2 (1.3)	6 (4.5)	< 0.001	0.09	< 0.001
	2 (1.0)	27 (5.5)	6 (4.0)	6 (4.5)			
	27 (13.4)	146 (30.0)	31 (20.8)	29 (21.8)			
	85 (42.1)	210 (43.1)	65 (43.6)	63 (47.4)			

**of home care patients**	86 (42.6)	97 (19.9)	45 (30.2)	29 (21.8)			
**8. Provide consultation to patients about their illness**	2 (1.0)	8 (1.6)	4 (2.6)	6 (4.5)	NA	< 0.001	< 0.001
	3 (1.5)	26 (5.3)	10 (6.6)	23 (17.3)			
	13 (6.4)	117 (24.0)	33 (21.9)	30 (22.6)			
	72 (35.5)	213 (43.7)	49 (32.5)	54 (40.6)			
	113 (55.7)	123 (25.3)	55 (36.4)	20 (15.0)			
**9. Provide consultation to patients about over-the-counter drugs**	1 (0.5)	4 (0.8)	4 (2.7)	5 (3.8)	< 0.001	< 0.001	< 0.001
	0 (0.0)	25 (5.1)	7 (4.7)	7 (5.3)			
	18 (8.9)	146 (30.0)	42 (28.0)	21 (15.8)			
	74 (36.5)	201 (41.3)	54 (36.0)	68 (51.1)			
	110 (54.2)	111 (22.8)	43 (28.7)	32 (24.1)			
**10. Provide consultation to patients about non-pharmaceutical health promotion (supplements, diet, and exercise)**	4 (2.0)	13 (2.7)	5 (3.3)	7 (5.3)	NA	< 0.001	< 0.001
	2 (1.0)	32 (6.6)	14 (9.3)	19 (14.3)			
	24 (11.8)	163 (33.5)	51 (34.0)	37 (27.8)			
	82 (40.4)	192 (39.4)	56 (37.3)	50 (37.6)			
	91 (44.8)	87 (17.9)	24 (16.0)	20 (15.0)			
**11. Provide online medication education to outpatients and home care patients**	1 (0.5)	3 (0.6)	0 (0.0)	2 (1.5)	0.06	0.46	0.51
	3 (1.5)	23 (4.7)	6 (4.0)	5 (3.8)			
	51 (25.2)	164 (33.7)	48 (32.0)	29 (21.8)			
	90 (44.6)	192 (39.4)	65 (43.3)	70 (52.6)			
	57 (28.2)	105 (21.6)	31 (20.7)	27 (20.3)			

**12. Store sufficient medicines at hospitals and pharmacies to prepare for a large-scale disaster**	3 (1.5)	0 (0.0)	0 (0.0)	1 (0.8)	< 0.001	1.00	1.00
	2 (1.0)	20 (4.1)	1 (0.7)	4 (3.0)			
	34 (16.7)	133 (27.3)	24 (16.0)	22 (16.5)			
	74 (36.5)	187 (38.4)	55 (36.7)	52 (39.1)			
	90 (44.3)	147 (30.2)	70 (46.7)	54 (40.6)			
**13. Recommend preventive medicine (e.g., lifestyle changes, hospital visits and medical checkups) to patients**	2 (1.0)	5 (1.0)	1 (0.7)	7 (5.3)	< 0.001	< 0.001	< 0.001
	0 (0.0)	17 (3.5)	8 (5.3)	13 (9.8)			
	18 (8.8)	160 (32.9)	32 (21.3)	26 (19.5)			
	82 (40.2)	197 (40.5)	72 (48.0)	51 (38.3)			
	102 (50.0)	108 (22.2)	37 (24.7)	36 (27.1)			
**14. Actively communicate with patients to establish better relationships**	0 (0.0)	5 (1.0)	0 (0.0)	1 (0.8)	< 0.001	< 0.001	< 0.001
	0 (0.0)	15 (3.1)	1 (0.7)	2 (1.5)			
	3 (1.5)	113 (23.2)	13 (8.7)	9 (6.8)			
	38 (18.7)	219 (45.0)	54 (36.0)	41 (30.8)			
	162 (79.8)	135 (27.7)	82 (54.7)	80 (60.2)			
**15. Obtain information on local hospitals and nursing homes**	2 (1.0)	3 (0.6)	0 (0.0)	3 (2.3)	< 0.001	0.038	1.00
	3 (1.5)	19 (3.9)	3 (2.0)	4 (3.0)			
	20 (9.9)	149 (30.6)	33 (22.0)	18 (13.5)			
	101 (49.8)	205 (42.1)	69 (46.0)	63 (47.4)			
	77 (37.9)	111 (22.8)	45 (30.0)	45 (33.8)			
